# Optical coherence tomography angiography biomarkers of microvascular alterations in RVCL-S

**DOI:** 10.3389/fneur.2022.989536

**Published:** 2022-08-26

**Authors:** Mays Al-Nofal, Irene de Boer, Seda Agirman, Anne E. Wilms, Amir H. Zamanipoor Najafabadi, Gisela M. Terwindt, Irene C. Notting

**Affiliations:** ^1^Department of Ophthalmology, Leiden University Medical Center, Leiden, Netherlands; ^2^Department of Neurology, Leiden University Medical Center, Leiden, Netherlands

**Keywords:** RVCL-S, biomarker, retina, cerebral small vessel disease, imaging, optical coherence tomography angiography

## Abstract

**Background:**

The brain and retina share many neuronal and vasculature characteristics. We investigated the retinal microvasculature in patients with a monogenic vasculopathy using optical coherence tomography angiography (OCTA). OCT-A is a novel precise non-invasive imaging method that may provide biomarkers suitable for diagnosis and follow-up of small vessel diseases.

**Methods:**

In this exploratory cross-sectional study, eleven RVCL-S patients and eleven age-matched healthy control participants were included. The size of the foveal avascular zone (FAZ) and the vascular density of the superficial capillary networks in the retina were measured by OCT-A.

**Results:**

The symptomatic and presymptomatic patients showed significantly lower vascular density values than controls in the foveal region [median (IQR) 18.2% (15.8–18.6) vs. 24.4% (21.5–26.8) (*p* < 0.001), 29.8% (29.6–30.8) vs. 33.2% (32.0–33.6) (*p* = 0.002), respectively]. The FAZ was significantly larger in the symptomatic RVCL-S patients than in the control group [13,416 square pixels [7,529–22,860] vs. 1,405 square pixels [1,344–2,470] (*p* < 0.001)]. No significant difference was identified in measurements of FAZ comparing presymptomatic and controls.

**Conclusion:**

Our findings with OCT-A demonstrated that RVCL-S causes an increase in the size of the FAZ in symptomatic RVCL-S patients compared to healthy participants. Moreover, there is a decrease in vessel density in the superficial capillary networks in both symptomatic and presymptomatic patients. In the future, newly developed precise objective instruments such as OCT (-A) may provide important tools in determining disease activity for follow up of common small vessel diseases.

## Introduction

Retinal Vasculopathy with Cerebral Leukoencephalopathy and Systemic manifestations (RVCL-S) is a monogenic small vessel disease caused by heterozygous C-terminal truncating mutations in *TREX1* (1). The best-known features of this disease are neurological manifestations of focal and diffuse brain dysfunction and white matter and intracerebral mass lesions on neuroimaging, and progressive blindness due to vascular retinopathy (2, 3). Besides these major features, RVCL-S can also be accompanied by a wide range of systemic manifestations including renal and liver disease, hypothyroidism and anemia (4). It is unknown how mutations in the C-terminus of TREX1 cause the multisystem vasculopathy seen in individuals with RVCL-S. Nevertheless, the endothelium appears involved in the pathogenesis of the disorder (5–7). RVCL-S serves as a monogenic model for common small vessel diseases and vascular brain disorders, such as vascular dementia.

Efficient early diagnostic testing of cerebral small vessel disease is still challenging and difficult. Therefore, the discovery of new non-invasive and economical screening tools for diagnosis is a major goal of current research. Due to the shared embryological origin of the retina and brain, the retina is regarded as an extension of the central nervous system, providing an opportunity to observe both neuronal and vascular changes in the brain. Optical coherence tomography angiography (OCT-A) is a novel non-invasive imaging technique that enables a detailed angiographic view of the retinal vascular network (8). In addition, as OCT-A does not require intravenous injections of a dye contrast, patients avoid most of the side effects that occur with conventional angiography.

OCT-A data on retinal vasculature may not only serve as an additional diagnostic parameter. A detailed analysis of the retinal vasculature could help understand its role in disease pathophysiology and provide biomarkers for treatment response, not only in retinal but also in neurological disorders. In Alzheimer's disease, vessel density has been shown to be decreased in the superficial capillary networks (9–11), and an enlarged foveal avascular zone (FAZ) has been described (10, 11). The FAZ is the round, capillary-free zone within the macula. FAZ area has been shown to correlate with cognitive performance, indicating its potential as a useful biomarker (12). In Parkinson's disease, another neurodegenerative disorder, there also appears to be a decreased vessel density in the retina (13). In another monogenic small vessel disease, cerebral autosomal dominant arteriopathy with subcortical infarcts and leukoencephalopathy (CADASIL), a decrease in vascular density in the retina has been demonstrated (14, 15).

Measurements of FAZ size and vascular density using OCT-A have been shown to be highly reproducible (16, 17). Moreover, measurements of the foveal avascular zone area and vessel density have proven to be indicative of microcirculatory problems of the retina. This exploratory study was designed to assess the potential of OCT-angiography in providing non-invasive biomarkers for RVCL-S. Our main aim was to demonstrate whether there were differences between symptomatic RVCL-S patients and healthy controls, as we expected the greatest difference between these groups. Secondly, we evaluated differences between presymptomatic patients and controls to determine the potential of OCT-A measurements as early biomarkers.

## Materials and methods

### Participants

This cross-sectional pilot study was conducted at the Leiden University Medical Center (LUMC) in the Netherlands, a tertiary referral center for RVCL-S, where all known families with RVCL-S from the Netherlands are seen. Symptomatic and presymptomatic RVCL-S patients with DNA proven mutations in the *TREX1* gene were recruited. Patients were considered symptomatic if they had any vascular abnormality such as micro-aneurysms, hemorrhages, focal and extended areas of ischemia or neovascular proliferation detected by fundoscopy or fluorescein angiography (FA) of the retina, neurological symptoms or RVCL-S specific findings on MRI (punctate T2 hyperintense white matter lesions with nodular enhancement; and/or larger T2 hyperintense white matter mass lesions with rim-enhancement, mass effect, and surrounding edema). Two age-matched control groups were selected out of an ophthalmological control cohort at the LUMC to achieve a similar age distribution. Exclusion criteria were history of diabetes mellitus, age-related macular degeneration, macular dystrophy, eye trauma or primary glaucoma. Controls were excluded if they had (a history of) ocular, systemic, metabolic, or cerebrovascular diseases. All participants were ≥18 years of age and gave informed consent, as documented in their medical file. The study was performed according to the tenets of the Helsinki Declaration and the protocol was approved by the Ethics Committee of the LUMC.

### Ophthalmological examination and OCT-A imaging

All patients underwent a detailed ophthalmologic examination by the same physician (MA), supervised by a neuro-ophthalmologist (ICN), including visual acuity measurement, slit lamp examination, applanation tonometry and fundoscopy. Intraocular pressure (IOP) was measured using Goldmann applanation tonometry after instillation of topical oxybuprocaine monofree 0.4% and fluorescein dye. After achieving a mydriatic effect using topical tropicamide monofree 0,5%, OCT-A scans (3x3 mm, focused on the central part of the macula), using NideK RS-3000 (NIDEK, Aichi, Japan) (for symptomatic and age-matched controls) and Heidelberg Spectralis (Heidelberg Engineering, Franklin, USA) (for presymptomatic and age-matched controls), were performed. One OCT-A scan was performed per eye per participant. The superficial capillary plexus was visualized, using the automatic software segmentation algorithm. The superficial capillary plexus was defined as the plexus between the ILM (Internal Limiting Membrane) and IPL (Inner Plexiform Layer) interface. The automatically generated OCT-A images were reviewed manually.

### OCT-A imaging processing

**Figure 1:**
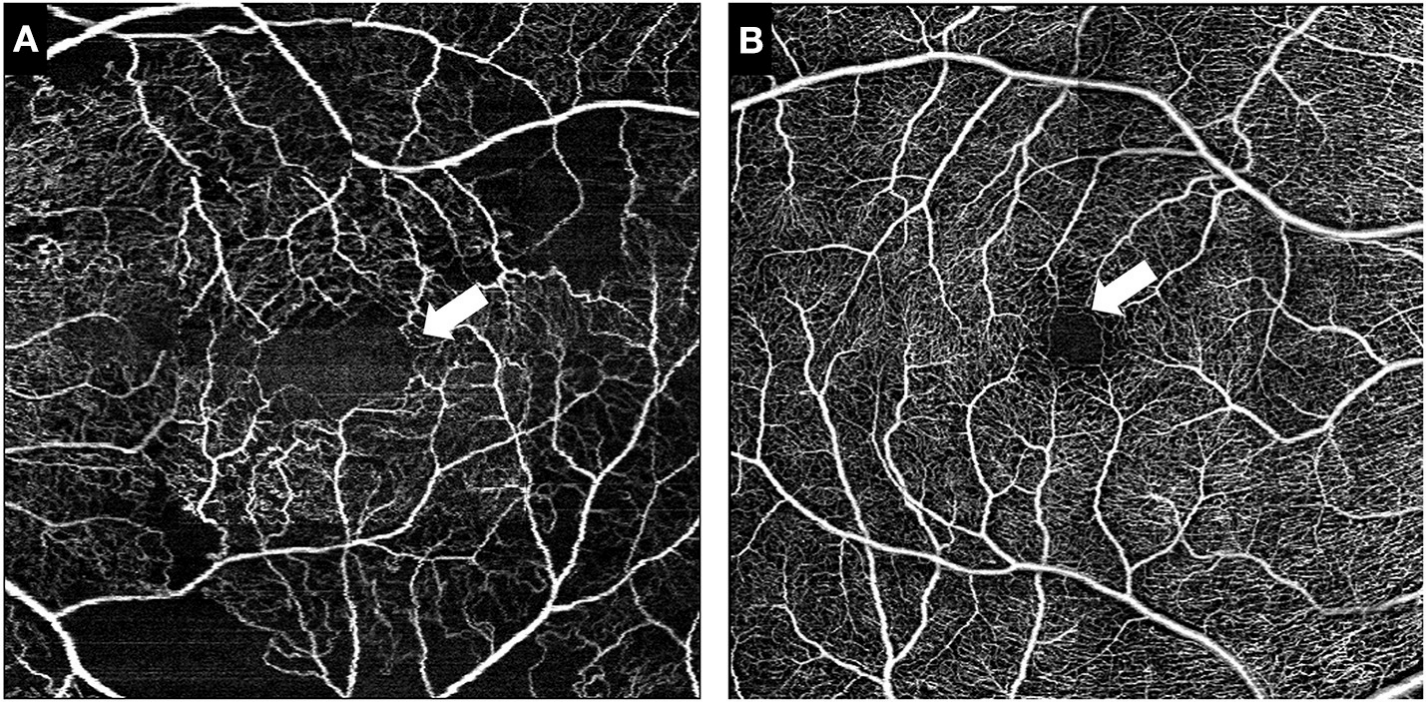
Representative images of the foveal avascular zone (FAZ). White arrow indicates the outlines of the FAZ in a symptomatic RVCL-S patient **(A)** and an age-matched healthy control **(B)**.

OCT-A image processing was performed in the Fiji ImageJ software (developed by Wayne Rasband MD, National Institutes of Health, Bethesda; available at https://imagej.nih.gov/ij/download.html) (Figure 1). The 3x3 mm images were adjusted to correspond with 512x512 pixels, setting the scale on 170,6667 pixels/mm. The area of the foveal avascular zone (FAZ) was outlined manually to automatically calculate the surface area. The vessel density of the superficial capillary networks was defined as the proportion of vessel area with blood flow over the total area measured. The vessel density was measured using a plugin feature on ImageJ (available at https://imagej.net/plugins/vessel-analysis), after transforming the image to a binary color code. Eyes were excluded if movement artifacts were present.

### Statistical analyses

Statistical analyses were performed using SPSS 25.0 (IBM Corporation, Armonk, NY). Demographics and OCT-A values are presented as median [interquartile range (IQR)]. FAZ size and vascular density were analyzed using generalized estimating equations (matrix: exchangeable) analyses to account for inter-eye correlations within participants. A *p*-value of <0.05 was considered significant.

## Results

### Demographics

Six symptomatic DNA proven RVCL-S patients (2 females and 4 males) (11 eyes) and 5 presymptomatic patients (3 female and 2 male) (10 eyes) were included in this study. In the RVCL-S group one patient was monocular due to enucleation following complications of neovascular glaucoma. In the presymptomatic group, in three eyes vascular density could not be determined due to incomplete image acquisition. Two age-matched control groups were included, one for the symptomatic (6 women) (10 eyes) and one for the presymptomatic RVCL-S group (3 females and 2 males) (10 eyes). In total three eyes (one symptomatic patient, two controls) were excluded due to movement artifacts on OCT-A images.

The median age in the RVCL-S group was 62.5 years (IQR 48.0–67.0). The median age in the group of healthy controls was 61.0 years (IQR 55.0–63.0). The median age in the presymptomatic RVCL-S group was 30 years (IQR 29.0–35.0) and the median age in the matched healthy control group was 25.0 years (IQR 23.0–31.0). The median visual acuity of the symptomatic RVCL-S patients was 0.0 in logMAR (IQR 0.0–0.2). Presymtomatic patients and controls had no visual impairments. Intra-ocular pressure was within normal limits in all participants. For a further clinical description of the patient cohort, see Table 1.

**Table 1:** Clinical findings in the RVCL-S cohort.

	**Symptomatic**	**Presymptomatic**
	**RVCL-S patients (*n* = 6)**	**RVCL-S patients (*n* = 5)**
Retinopathy [yes, *n* (%)]	6/6 (100)	0/0 (0)
Features of focal or global brain dysfunction [yes, *n* (%)]	4/6 (67)	0/0 (0)
RVCL-S specific MRI findings [yes, *n* (%)]^a^	4/5 (80)	0/0 (0)
Internal organ dysfunction [yes, *n* (%)]^b^	3/6 (50)	0/0 (0)

^a^Of one patient no MRI was available. ^b^Internal organ dysfunction was defined as organ dysfunction (liver, kidney and thyroid disease and anemia) requiring treatment.

### OCT-A imaging in symptomatic RVCL-S patients vs. controls

**Figure 2:**
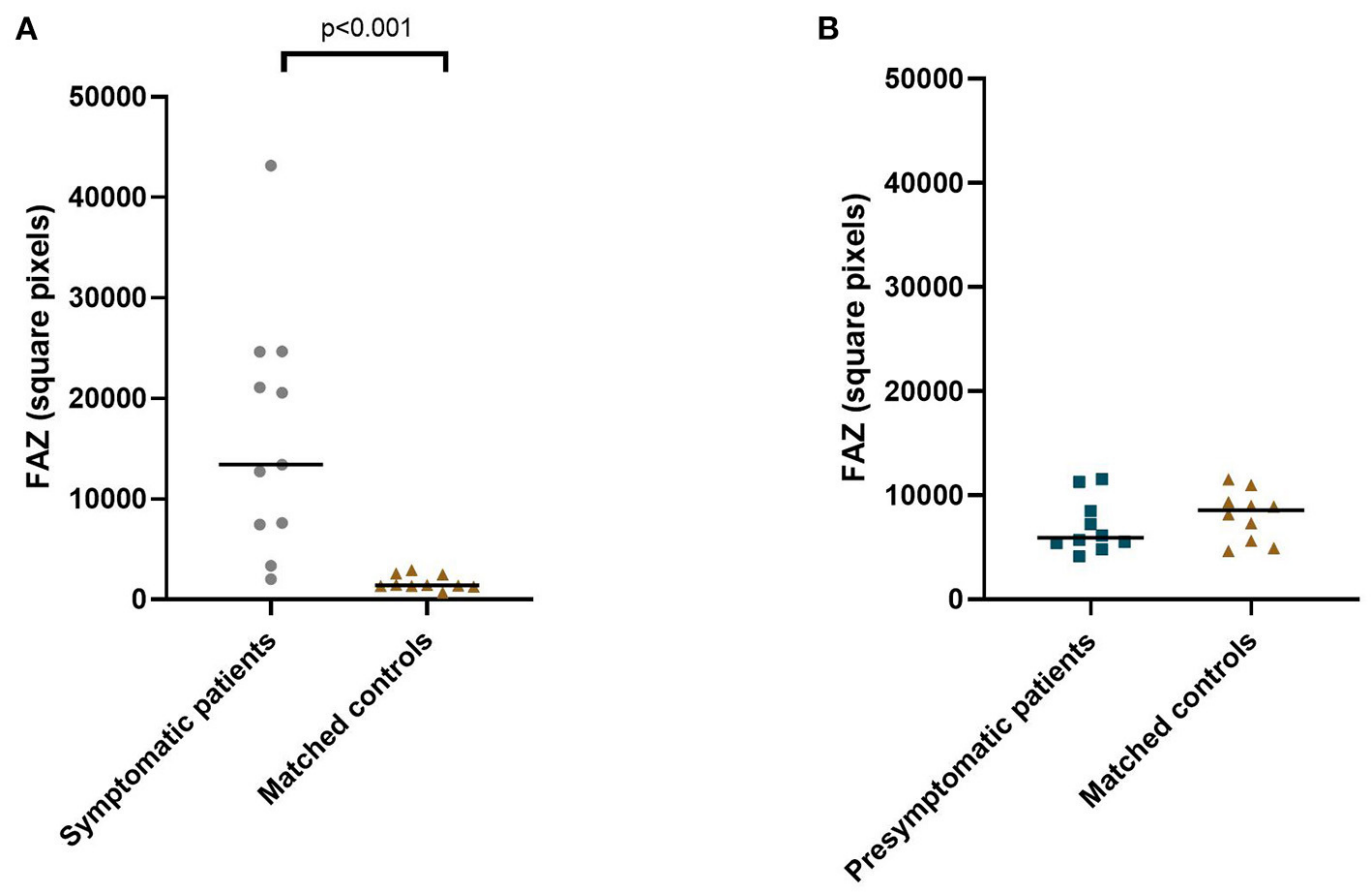
Median foveal avascular zone area in symptomatic RVCL-S patients and controls **(A)** and presymptomatic RVCL-S patients and controls **(B)**.

**Figure 3:**
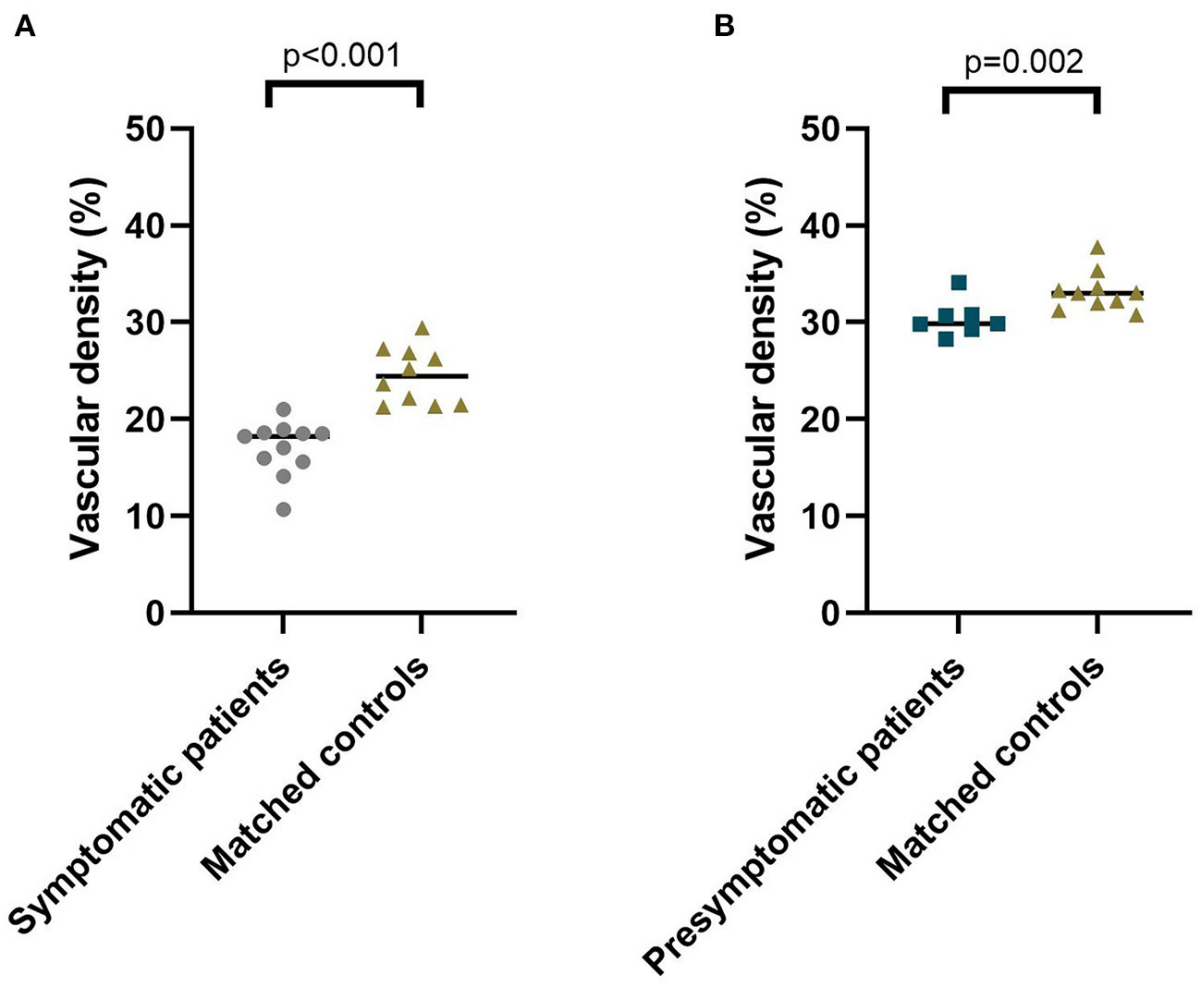
Median vascular density of the superficial capillary networks in symptomatic RVCL-S patients and controls **(A)** and presymptomatic RVCL-S patients and controls **(B)**.

The area of the foveal avascular zone was greater in symptomatic RVCL-S patients when compared to controls [median (IQR) 13,416 square pixels (7,529–22,860) vs. 1,405 square pixels (1,344–2,470), *p* < 0.001] (Figures 1, 2A, Table 2). Moreover, vessel density in the superficial capillary networks was decreased in symptomatic RVCL-S patients [median (IQR) patients: 18.2% (15.8–18.6) vs. controls: 24.4% (21.5–26.8), *p* < 0.001] (Figure 3A, Table 2).

**Table 2:** Foveal avascular zone area and vascular density in RVCL-S patients vs. controls.

	**Co-efficient**	**CI**	***P*-value**
**Symptomatic patients**			
Foveal avascular zone area	14,734.4	7,940.8–21,528.0	<0.001
Vessel density	−7.5	−9.8 to −5.1	<0.001
**Presymptomatic patients**			
Foveal avascular zone area	−1,010	−3.0959–1,075.9	0.34
Vessel density	−2.8	−4.6 to −1.1	0.002

### OCT-A measurements as early biomarkers for RVCL-S: Presymptomatic patients vs. controls

No difference was found in FAZ size between presymptomatic RVCL-S patients and controls (Figure 2B, Table 2). The vessel density did appear to be decreased in presymptomatic RVCL-S patients [median (IQR) 29.8% (29.6–30.8) vs. 33.2% (32.0–33.6), *p* = 0.002] (Figure 3B, Table 2).

## Discussion

We discovered that the macular vessel density in the superficial capillary networks was lower in symptomatic and presymptomatic RVCL-S patients than in the healthy control groups. Moreover, we found that symptomatic RVCL-S patients had an increase in FAZ size compared to healthy participants. The eye can be used as a window into neurological conditions. As cerebral and retinal arterioles share a similar anatomy and embryology, there is evidence for an association between retinal vessel changes and cerebral small vessel disease (18, 19). OCT-A is a non-invasive technique that can be used as a tool to learn more about these vessels, but also to find biomarkers of disease onset or progression. Furthermore, architecture of retinal vasculature can provide essential clinical information not just in ocular disease but also in systemic vascular disorders. Given the pressing need for improved biomarkers in systemic vascular diseases, non-invasive imaging of retinal vessels appears a promising avenue of research.

In this study we compared manual measurements of the foveal avascular zone and the macular vascular density in the superficial capillary networks in RVCL-S patients with healthy controls. This pilot study already demonstrated retinal abnormalities found on OCT-A images in this small vessel disease. While we could not evaluate whether OCT-A findings correlated with symptomology in this pilot study, in CADASIL, another monogenic small vessel disease, macular vascular density correlated with gait speed, suggesting its use as a biomarker of advanced CADASIL (15). OCT-A findings are also a promising biomarkers in other neurodegenerative disorders. In preclinical Alzheimer Disease, patients without any visible abnormalities on fundoscopy already demonstrated an increased FAZ (20). Together, this underlines the possible clinical importance of OCT-A for rare and common neurovascular disorders.

Strikingly, in this pilot study we already demonstrated reduced vascular density in presymptomatic patients. A previous study in patients with cerebral small vessel disease also reported a decrease in vascular density, while no differences in FAZ size were demonstrated (21). The decrease in vascular density could therefore be the first sign of failure of autoregulation within the retinal circulation. As the blood-retinal-barrier (BRB) shares many similarities with the blood-brain-barrier (BBB) (endothelial cells, basement membranes, pericytes, glial cells and both are crucial for autoregulation) (19), similar pathological processes are likely responsible for both the retinal microvascular changes and the cerebral microangiopathy seen in cerebral small vessel diseases. Ischemic processes, inflammation and endothelial dysfunction have all been implicated to influence BRB and BBB function (19, 22). Interestingly, in patients with RVCL-S, impaired cerebrovascular reactivity, i.e., the ability of the cerebral vessels to dilate and constrict to vasoactive stimuli, has been demonstrated. While less pronounced, these findings were already present in mutation carriers younger than 40 years (7). Failure in regulation of blood flow could thus be a major component to the disease pathway and therefore, more research is needed to elucidate the mechanism behind these microvascular changes.

Our exploratory study has several limitations. Due to the small sample size we could not estimate the exact relationship between disease severity and the FAZ. Moreover, confirmatory studies with larger sample sizes are needed. Unfortunately, presymptomatic patients (and their age-matched) controls were included after another OCT-A device had become in use. Due to this use of two different OCT-A devices, we could not compare the processed OCT-A images of symptomatic with presymptomatic patients. In a follow-up study, this should be avoided when possible. Larger sample size will also be required for this comparison to be able to take into account the effect of age on OCT-A parameters. Another limitation was the impossibility to fixate for some participants. Due to the poor eye fixation in both the RVCL-S and the healthy control group (caused by patient related fatigue, poor visual acuity or limited physical condition) movement artifacts on the OCT-A images led to exclusion. However, this non-invasive imaging method could be very useful in patients with limited systemic symptoms.

A major strength of our study is the use of a unique hereditary neurovascular small vessel model for small vessel disease and vascular dementia. In this model we can study different disease stages in mutation carriers, which provides the unique opportunity to evaluate the presymptomatic disease stage. Also, to our knowledge, this is the first study describing the use of OCT-angiography in RVCL-S.

Recently, RVCL-S was shown to be associated with retinal thinning in the peripapillary and macular area (23). This already demonstrates the possible value of OCT findings as potential biomarkers for RVCL-S. Our pilot study indicates a possible added value of also measuring the foveal avascular zone and vascular density. A more extended study with a larger sample size, as well as a follow-up design, is needed to determine the exact characteristics and cut-off rates needed for the foveal avascular zone and vessel density to be used as biomarkers and to demonstrate their usefulness in clinical practice. Moreover relating OCT-A findings to MRI brain imaging markers will help further determine the role of OCT-A measurements as biomarkers. This promising first step toward achieving reliable retinal vascular biomarkers for RVCL-S, a monogenic small vessel disease, can also help elucidate biomarkers for common (cerebral) small vessel diseases.

## Conclusions

Using OCT-A, we demonstrated that RVCL-S leads to an increase in the size of the FAZ. Furthermore, there was a decrease in vessel density in the superficial capillary networks, which was already present in presymptomatic patients. In the future, newly developed precise objective instruments such as OCT (-A) may provide important tools in determining disease activity for follow up of RVCL-S, but possibly also of common small vessel diseases.

## Data availability statement

The raw data supporting the conclusions of this article will be made available by the authors, without undue reservation.

## Ethics statement

The studies involving human participants were reviewed and approved by Medisch-Ethische Toetsingscommisie Leiden Den Haag Delft. Written informed consent for participation was not required for this study in accordance with the national legislation and the institutional requirements.

## Author contributions

MA-N, IB, IN, and GT conceived and designed the study and wrote the first version of the manuscript. MA-N, SA, AW, and AZ collected the data. IB performed the analyses. All authors interpreted the data, provided intellectual input, revised the manuscript, and approved the manuscript before submission.

## Funding

This study was supported by Grants of International Retinal Research Foundation (IRRF) to GT, IN, and IB, Dioraphte (20010407 to GT and IN), and the Dutch Heart Foundation (2020T065 to IB). The funding organizations had no role in the design or conduct of this research.

## Conflict of interest

Author IB reports independent research support from the international retinal research foundation (IRRF) and Dutch Heart foundation. Author IN reports independent research support by the IRRF and Stichting Dioraphte. Author GT reports grants or consultancy support from Novartis, Lilly, Teva, Allergan, and independent support from Netherlands Organization for Health Research and Development (NWO, and ZonMW), NIH, European Community, Dutch Heart Foundation, and Dutch Brain Foundation, IRRF and Stichting Dioraphte. The remaining authors declare that the research was conducted in the absence of any commercial or financial relationships that could be construed as a potential conflict of interest.

## Publisher's note

All claims expressed in this article are solely those of the authors and do not necessarily represent those of their affiliated organizations, or those of the publisher, the editors and the reviewers. Any product that may be evaluated in this article, or claim that may be made by its manufacturer, is not guaranteed or endorsed by the publisher.
